# Decreasing Total Fertility Rate in Developing Countries 

**Published:** 2016-12

**Authors:** Mohammad Eslami

**Affiliations:** Population, Family and School Office, Ministry of Health and Medical Education, Tehran, Iran

Respected editor 

In his article Bellieni mentioned ‘in western countries couples have high expectative about the advantages of delayed parenthood and exploit fertility as late as possible’ (1).

Decreasing total fertility rate (TFR) is the number of children who would be born per woman (or per 1,000 women) if she/they were to pass through the childbearing years bearing children according to a current schedule of age-specific fertility rates. Replacement level is a figure of TFR and less than TFR it results in negative population growth rate, aging and population reduction. TFR below replacement level is an important problem in most developed and some developing countries (2). 

Iran experienced TFR below replacement level from 2006 (1.85). Results of next census (2011) revealed more decline in the country TFR (1.8) and it has been continued to be under replacement level. They were alarms to the policymakers to change country population policies in such a way to compensate this reduction. According to this warnings, new population policies developed and the Supreme Leader ratified population policy decree in 2014 (3).

There are very important items in this decree which some of them have direct effect on health and family health. They are include: eliminate obstacle to marriage, reduction in marriage age, allocate adequate resources for women especially during pregnancy and lactation, development and strengthening the social security system, and prevention and treatment in line with reproductive health and childbearing and so on (4).

In a national survey among Iranian girls and boys immediately before marriage, the number of wanted children calculated 2.3. Hopefully in case of achievement to this figure, the TFR below replacement level will be compensated. But why the total TFR in Iran is less than 2.1? There are some different reasons. Some of them are as below:

Age of marriage among Iranian girls and boys is in the increasing trend and it has been increased to 23.4 for girls and above 27 for boys (5). It may be considered as an important cause for decreasing the number of children among Iranian families. The item number two in the population policy decree address this important item.

After postponing the marriage, the mean interval between marriage and first child calculated more than 3.5 years (6). It means the married women bring their first child about 27 years old. It should be noted it is the average age for being the mother at the first time. Comparing to the previous decade(s) it is too late, actually as they will miss the best age interval for safe pregnancy and childbearing.

The mean interval between the first and second child is more than 5.5 years (6). It means, in case of bringing the second child among Iranian women, their mean age will be more than 32 years old and missing the best period for childbearing (20-30) actually will be happened. By age increasing, families will experience more concerns for the next pregnancies and it may cause to more contraception. These two above items has been addressed in item four of population policy decree under strengthening of reproductive health and childbearing services. Providing correct and unbiased reproductive health information in line with qualified services will be one of the most important approaches to increase society reproductive health literacy to adopt the best behavior for reproductive health.

Social securities, career, health and child expenditure are important reasons for late first pregnancies and long and unsafe intervals between the next pregnancies. Item three in population policy decree addressed these important items directly.

Infertility is one of the other important problems which may contribute the TFR below replacement level. According to a survey Iran is experiencing the infertility with a rate of about 20.2 percent (7). Postponing the marriage and late first pregnancies with this high figure of infertility will result in later treatments for infertility and reduction in treatment success. Item three in Supreme Leader population policy decree addresses the infertility problems directly.

Iran hope with such a comprehensive population policies and inter sectored collaboration (which is necessary for implementing of Population policy decree) achieve fertility replacement level and improving maternal, newborns and children health.

## References

[1] Bellieni C (2016). Pregnancy and Undue Pressures. J Fam Reprod Health.

[2] http://www.cpc.unc.edu/measure/prh/rh_indicators/specific/fertility/total-fertility-rate.

[3] http://www.leader.ir/fa/content/11847.

[4] Overall population policies by the supreme leader of Islamic revolution, Ayatollah Sayyid Ali Khamenei, 20 May 2014, Ministry of Science, Research and Technology, National population studies & Comprehensive management institute National census results 2011.

[5] http://iran.unfpa.org/Documents/Census2011/2011%20Census%20Selected%20Results%20-%20Eng.pdf.

[6] Rashidian A, Karimi-Shahanjarini A, Khosravi A, Elahi E, Beheshtian M, Shakibazadeh E (2014). Iran’s Multiple Indicator Demographic and Health Survey - 2010: Study Protocol. International Journal of Preventive Medicine.

[7] Akhondi MM, Kamali K, Ranjbar F, Shirzad M, Shafeghati S, Behjati Ardekani Z (2013). Prevalence of Primary Infertility in Iran in 2010. Iranian Journal of Public Health.

